# Induction of protective immune response against both PPRV and FMDV by a novel recombinant PPRV expressing FMDV VP1

**DOI:** 10.1186/1297-9716-45-62

**Published:** 2014-06-04

**Authors:** Chunsheng Yin, Weiye Chen, Qianqian Hu, Zhiyuan Wen, Xijun Wang, Jinying Ge, Qianqian Yin, Haibing Zhi, Chun Xia, Zhigao Bu

**Affiliations:** 1 Department of Microbiology and Immunology, College of Veterinary Medicine, China Agricultural University, Beijing 100094, China; 2 Key Laboratory of Veterinary Public Health of Ministry of Agriculture and State Key Laboratory of Veterinary Biotechnology, Harbin Veterinary Research Institute of Chinese Academy of Agricultural Sciences, Harbin 150001, China; 3 China Institute of Veterinary Drug Control, Beijing 100081, China; 4 College of Animal Science, Anhui Science and Technology University, Fengyang 233100, China

## Abstract

Peste des petits ruminants (PPR) and foot-and-mouth disease (FMD) are both highly contagious diseases of small domestic and wild ruminants caused by the PPR virus (PPRV) and the FMD virus (FMDV). In this study, a recombinant PPRV expressing the FMDV VP1 gene (rPPRV/VP1) was generated and FMDV VP1 expression did not impair replication of the recombinant virus in vitro and immunogenicity in inducing neutralizing antibody against PPR in goats. Vaccination with one dose of rPPRV/VP1 induced FMDV neutralizing antibody in goats and protected them from challenge with virulent FMDV. Our results suggest that the recombinant PPRV expressing the FMDV VP1 protein is a potential dual live vectored vaccine against PPRV and FMDV.

## Introduction

Foot and mouth disease (FMD) is a severe, highly contagious, clinically acute, economically devastating viral disease of wild and domestic cloven-hoofed animals, such as cattle, pigs, goats, and sheep. It is caused by foot and mouth disease virus (FMDV), a member of the family *Picornaviridae*, genus *Aphthovirus*. FMDV contains a 7–8-kb, single-stranded, positive-sense RNA genome that encodes a single polyprotein, which is cleaved into four structural proteins (viral protein (VP)4, VP2, VP3, and VP1) and eight non-structural proteins (L, 2A, 2B, 2C, 3A, 3B, 3C, and 3D polymerase). The genome is enclosed within a nonglycosylated, icosahedral capsid comprising 60 copies each of VP1–4 [1]. Seven immunologically distinct serotypes (A, O, C, SAT1, SAT2, SAT3, and Asia1) were identified on the basis of a VP1 coding region sequence. VP1 is exposed on the surface of viral particles and is the main antigen to elicit a neutralizing antibody response [2,3]. Currently, FMD is mainly controlled by inactivated virus vaccines, which provide only short-term protection (4–6 months), and may create carrier animals [4,5].

Peste des petits ruminants (PPR) is a highly contagious disease of domestic and wild small ruminants caused by the peste des petits ruminants virus (PPRV) and is responsible for serious socioeconomic problems in some of the poorest developing countries [6-8]. PPR was first reported in the Ivory Coast in 1942 and later found in the Middle and Near East, southwest and central Asia [9-12], and recently in China [13]. PPRV, which is a member of the genus *Morbillivirus* belonging to the family *Paramyxoviridae*[14], contains a linear, non-segmented, single-stranded, negative-sense, 15948-bp, RNA genome, which encodes six structural proteins, nucleocapsid (N), phosphoprotein (P), matrix (M), fusion (F), hemagglutinin (H) and polymerase (L), and two nonstructural proteins, C and V that are genomically encoded in the order of 3′-N-P/C/V-M-F-H-L-5′ [15,16]. An attenuated PPRV strain, Nigeria/75/1, derived from serial-passage in Vero cells, has been widely used as a safe and efficacious live vaccine to control PPR infections [17].

Several studies have indicated that recombinant paramyxoviruses are effective and genetically stable vectors with many advantages. It is easy to generate recombinant paramyxovirus vectored vaccine candidates using reverse genetic techniques, the recombinant vaccine could be used to prevent two diseases to reduce the costs, and it could potentially be used as a DIVA (Differentiating Infected from Vaccinated Animals) vaccine [18-20]. Rinderpest virus, which is also a member of the genus *Morbillivirus*, has been used as vector expressing FMDV epitopes [21]. In our previous study [22], reverse genetics for PPRV was successfully established, providing a novel method to develop bivalent, live-vectored vaccines against PPRV and other important viral diseases in goats and sheep, such as FMDV, Rift valley fever, and bluetongue virus. In this study, we constructed a recombinant PPRV expressing FMDV VP1 and its immunogenicity against PPRV and FMDV was evaluated in goats.

## Material and methods

### Viruses and cells

Live PPRV attenuated vaccine strain Nigeria 75/1 (N75/1) was obtained from the China Institute of Veterinary Drug Control (Beijing, China) who imported it from The Pirbright Institute (Pirbright, United Kingdom). BHK-21 cells (ATCC no.: CCL-10; The American Type Culture Collection, Manassas, VA, USA) and Vero cells (ATCC no.: CCL-81) were cultured in Dulbecco’s modified Eagle’s medium (DMEM; Gibco, Carlsbad, CA, USA) containing 10% fetal bovine serum (FBS) (Gibco). N75/1 and rescued recombinant PPRV were propagated and titrated in Vero cells cultured in DMEM containing 2% FBS. FMDV strain Asia1/JSL/GSZY/06 (JSL/06) was conserved in Baoshan Bio-pharmaceutical Factory of China Animal Husbandry Industrial Co., Ltd. (Baoshan, China) and propagated in 2–7-day old suckling mice. A 50% goat infective dose was determined in goats [23].

### Plasmid construction and rescue of recombinant viruses

**Figure 1:**
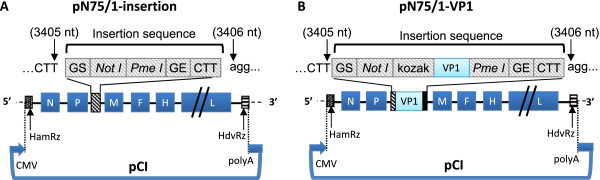
**Plasmid construction for recombinant PPRV rescue. (A)** pN75/1-insertion was constructed previously [22] through insertion of a morbillivirus gene start (GS) sequence, *Not* I and *Pme* I sites, gene end (GE) sequence, and CTT intergenic trinucleotides between the P and M genes of genomic PPRV cDNA. **(B)** The VP1 ORF with a Kozak sequence at the 5*'* end of the ORF was inserted into plasmid pN75/1-insertion to generate plasmid pN75/1-VP1.

The plasmid pN75/1 insertion (Figure 1A) contained a full-length N75/1 cDNA sequence flanked with a hammerhead ribozyme sequence (HamRz), hepatitis delta virus ribozyme sequence, and insertion sequence (a gene start sequence, *Not* I and *Pme* I restriction sites, a gene end sequence, and an intergenic CTT trinucleotide), and helper plasmids (pCA-N, pCA-P and pCA-L) were constructed as previously described [22]. The cDNA for the open reading frame (ORF) of the FMDV VP1 (Asia1) protein was synthesized according to a published sequence (GenBank accession no.: GU931682). The *Not I* restriction sequence (bold), Kozak sequence (gccgccacc, low case and italic) and the ATG initiation codon were introduced at the 5′ end of the cDNA encoding VP1; the TAA termination codon and *Pme* I restriction sequence (uppercase and italic) were introduced at the 3′ end of the cDNA encoding VP1, and the final DNA fragment (**GCGGCCGC***gccgccacc*ATGactaccaccact……cctgagaaacagTAA*GTTTAAAC*) was treated with *Not I and Pme*I and inserted into the plasmid pN75/1 insertion between *Not I and Pme*I sites. The resulting pN75/1-VP1 plasmid was used to rescue the recombinant virus. The method to rescue the recombinant virus was described previously [22]. Briefly, 90% confluent Vero cells in one well of a 6-well plate were transfected with the plasmids pCA-N (2 μg), pCA-P (1 μg), and pCA-L (1 μg) together with 4 μg of pN75/1-VP1. Lipofectamine 2000 reagent (Invitrogen, Carlsbad, CA, USA) was used for transfection following the manufacturers’ instructions. After 7–9 days of incubation at 37 °C, the cells and supernatants were collected and freeze-thawed twice and then passaged in fresh cells to propagate the rescued viruses. Supernatants from cytopathic effect-positive wells were used to propagate viral stocks in Vero cells. The complete genomic sequences of the rescued viruses were confirmed by sequencing. The rescued virus was named rPPRV/VP1.

### Immunofluorescence assay (IFA)

Vero cells grown in 24-well plates were infected with N75/1 or rPPRV/VP1 at a multiplicity of infection (MOI) of 0.1 and incubated for 3 days. The cells were fixed with 3% paraformaldehyde in phosphate-buffered saline and stained with anti-N75/1 mouse serum [24,25] or anti-FMDV VP1 rabbit serum (Asia1 type) [26] followed by tetramethyl rhodamine isothiocyanate-labeled goat anti-mouse immunoglobulin IgG (Sigma-Aldrich, St. Louis, MO, USA) or fluorescein isothiocyanate-labeled goat anti-rabbit IgG (Sigma). Mock-infected cells were used as controls. The fluorescence was observed using an inverted fluorescence microscope (Carl Zeiss AG, Oberkochen, Germany).

### Western blotting

Vero cells were infected with N75/1 or rPPRV/VP1 at an MOI of 0.1 and incubated for 5 days, and BHK-21 cells were infected with FMDV JSL/06 at an MOI of 0.1 and incubated for 12–16 h. The N75/1 and rPPRV/VP1 particles were both purified by sucrose gradient centrifugation with 60%, 40% and 20% density (140 000 *g*). The cell extracts of Vero and BHK-21 and purified virus particles were analyzed by sodium dodecyl sulfate polyacrylamide gel electrophoresis (SDS-PAGE) and blotted onto a nitrocellulose membrane, which was then incubated with anti- FMDV-VP1 rabbit serum (Asia1 type) [26] or anti-PPRV-N rabbit serum produced through immunization with purified recombinant PPRV N expressed in E.coli as the first antibody, and horseradish peroxidase-conjugated goat anti-rabbit IgG (Sigma-Aldrich) as the secondary antibody. Immunostained proteins were visualized with 3,3′-diaminobenzidine reagent. Mock-infected Vero cells and mock-infected BHK-21 cells were used as controls.

### Vaccination and viral neutralizing antibody (NA) assay

One-year-old black goats (a local breed of Yunna Province, China) without neutralizing antibodies to FMDV (titre < 8) and PPRV (titre < 5) were immunized by intramuscular injection at the neck with a 50% tissue culture infective dose (TCID_50_) of 6 × 10^6^ rPPRV/VP1 or N75/1. Sera were collected at 14, 21, 28, and 40 days post-vaccination for NA assays. The NA to PPRV N75/1 were titrated in Vero cells as described previously [22,24] and the NA to FMDV JSL/06 was titrated in BHK-21 cells following the protocol recommended by the World Organization for Animal Health (Office International des Epizooties) [23]. Antibody titers were expressed as the reciprocal of the final dilution of serum in the serum/virus mixture which neutralized an estimated 100 TCID_50_ of virus at the 50% end-point [27].

### Challenge study

Goats vaccinated with N75/1 or rPPRV/VP1 were transferred from a normal sheepfold to a level 3 animal facility, where the animals were acclimated for 1 day before viral challenge. Each goat was challenged with virulent FMDV JSL/06 at 40 days post-vaccination by two intradermal injections to the tongue (0.1 mL at each point; a total of 1000 goat infectious dose _50_ of FMDV for each goat). The animals were observed for 14 days post-challenge. Rectal temperature (°C) was measured daily and heparinized blood and oropharyngeal swabs were collected at different days for FMDV detection by inoculation of BHK-21 cells [28]. Simultaneously, lesions and clinical signs were observed and evaluated daily and scored as follows:

0: no water vacuole or ulceration appeared.

1: a water vacuole or ulceration was only found at the injection point.

2: one water vacuole or ulceration was found on the tongue surface, rhinarium, gingiva, or lips, but not at the injection point.

3: two or more water vacuoles or ulcerations were found on the tongue surface, rhinarium, gingiva, or lips, but not at the injection point, or water vacuoles or ulcerations were found at one ungula.

4: two or more water vacuoles or ulcerations were found on the tongue surface, rhinarium, gingiva, or lips, but not at the injection point, while water vacuoles or ulcerations were found at one or more ungulas.

### Statistical analysis

Statistical analyses were performed by two-way analysis of variance (ANOVA) or *t*-tests using GraphPad Prism statistical software (GraphPad Software, Inc., La Jolla, CA, USA). A probability (*p*) value < 0.05 was considered statistically significant, and < 0.01 statistically very significant.

## Results

### Generation of recombinant PPRV expressing FMDV VP1

**Figure 2:**
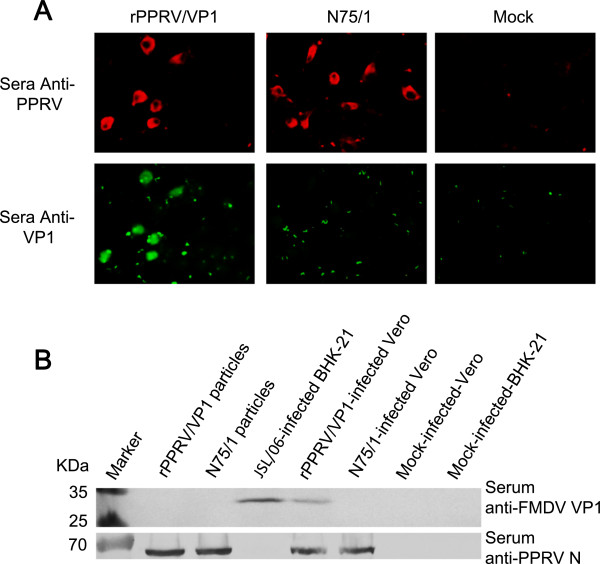
**VP1 protein replication and expression of rPPRV/VP1 in Vero cells. (A)** Cells infected with rPPRV/VP1, N75/1, or mock-infected were fixed and labeled with anti-N75/1 mouse serum for the presence of PPRV protein (red) or anti-FMDV VP1 (Asia-I type) rabbit serum for the presence of the VP1 protein (green). **(B)** The N75/1 and rPPRV/VP1 particles were respectively purified by sucrose gradient centrifugation with 60%, 40% and 20% density (140 000 *g*). Lysates of N75/1- or rPPRV/VP1-infected Vero cells, lysates of FMDV JSL/06-infected Vero cells, N75/1 particles and rPPRV/VP1 particles were respectively probed by SDS-PAGE and western blotting using anti-FMDV VP1 (Asia-I type) rabbit serum and anti-PPRV-N rabbit serum respectively. Mock-infected Vero and BHK-21 cells were used as controls.

To construct a recombinant PPRV expressing the FMDV VP1 protein (rPPRV/VP1), the FMDV VP1 gene was inserted in the genome cDNA of PPRV N75/1 (Figure 1A) between the P and M genes (Figure 1B). VP1 gene insertion in the genome of rescued recombinant virus rPPRV/VP1 was confirmed by reverse transcriptase polymerase chain reaction (RT-PCR) (data not shown) FMDV VP1 expression in rPPRV/VP1-infected Vero cells was confirmed by IFA. Both rPPRV/VP1- and N75/1-infected Vero cells were positive to anti-N75/1 mouse serum [24,25] by IFA (Figure 2A). rPPRV/VP1-infected Vero cells were positive, while N75/1-infected Vero cells were negative to rabbit serum anti-FMDV(Asia1 type) VP1 (Figure 2A). A 28-kDa band representing the FMDV VP1 protein was detected by western blotting with rabbit serum anti-FMDV(Asia1 type) VP1 in rPPRV/VP1-infected Vero cells and JSL/06-infected BHK-21 cells, but not in mock-infected Vero cells, N75/1-infected Vero cells, mock infected-BHK-21 cells, purified rPPRV/VP1 particles and N75/1 particles (Figure 2B). At the same time, a 58-kDa band representing the PPRV N protein was detected with rabbit serum anti-PPRV N in rPPRV/VP1-infected vero cells, N75/1-infected Vero cells, purified N75/1 particles and rPPRV/VP1 particles, but not in JSL/06-infected BHK-21 cells, mock-infected-Vero cells and mock-infected-BHK-21 cells.

**Figure 3:**
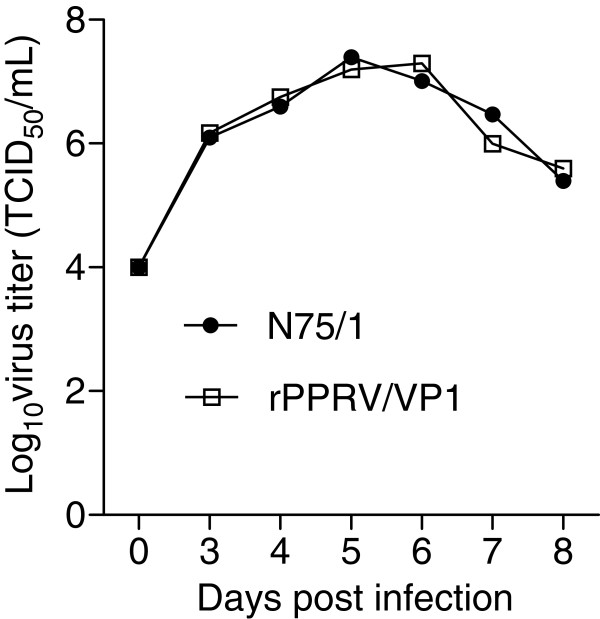
**Growth of rescued viruses in Vero cells.** The viral titers in rPPRV/VP1- or N75/1-infected Vero cells were measured at different days post infection.

To determine whether the foreign gene insertion affected the replicative ability of the vector virus, growth curves for rPPRV/VP1- and N75/1-infected Vero cells were determined and compared. The TCID_50_ was quantitated using previously described methods [29]. The results show no significant differences in growth titer at different times post-infection between the two viruses (Figure 3). The genetic stability of the FMDV VP1 gene within rPPRV/VP1 was assessed by serially passaging the virus for 10 times in Vero cells. After 10 passages, the presence of a genomic FMDV VP1 sequence was confirmed by RT-PCR and genome sequencing (data not shown). FMDV VP1 expression in rPPRV/VP1- infected Vero cells was confirmed by IFA and western blotting (data not shown).

### rPPRV/VP1 induced FMDV and PPRV NA in goats

**Figure 4:**
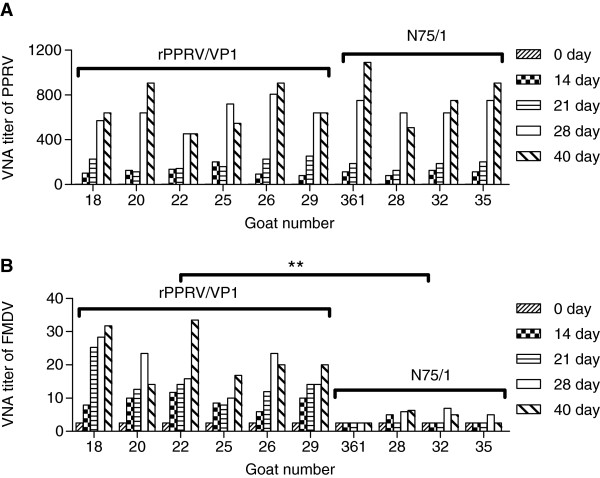
**Viral NA responses elicited by rPPRV/VP1 and N75/1 in goats.** Six goats (nos. 18, 20, 22, 25, 26, and 29) were inoculated by intramuscular injection with rPPRV/VP1 and four goats (nos. 361, 28, 32 and 35) were inoculated by intramuscular injection with N75/1. The sera were collected at 14, 21, 28, and 40 days post-inoculation and were tittered for PPRV NA **(A)** and FMDV NA **(B)**. The data were analyzed by the two-way ANOVA method using the GraphPad Prism statistical software (**, *p* < 0.01).

To evaluate the immunogenicity of the recombinant virus, sera were collected from six rPPRV/VP1-vaccinated goats and four N75/1-vaccinated goats at 14, 21, 28, and 40 days post-vaccination and subjected to the NA assay for FMDV and PPRV. As shown in Figure 4A, all animals underwent serum conversion to PPRV (NA titer ≥ 10) by 14 days post-vaccination and PPRV NA titers gradually increased until 40 days post-vaccination. The NA titer to PPRV ranged from 500 to 800 and 500 to 1100 at 28 and 40 days post-vaccination, respectively. There was no statistically significant difference in PPRV VN titers between rPPRV/VP1-vaccinated goats and N75/1-vaccinated goats at different days post-vaccination (*p* > 0.05). rPPRV/VP1 also induced significant FMDV-specific NA responses. At 14 days post-vaccination, five of six animals vaccinated with rPPRV/VP1 showed FMDV VN titers ≥ 8. Afterward, FMDV NA titers continually increased. At post-vaccination days 28 and 40, FMDV NA titers in all six animals were 11–28 and 14–34, respectively. As the control, all four N75/1-vaccinated animals showed post-vaccination FMDV NA titers of < 8 (Figure 4B). There was a statistically significant difference in FMDV VN titers between rPPRV/VP1- and N75/1-vaccinated goats at different days post-vaccination (**, *p* < 0.01). The temperature of all the animals inoculated with rPPRV/VP1 or N75/1 remained normal after vaccination.

### rPPRV/VP1 protects goats from virulent FMDV challenge

**Figure 5:**
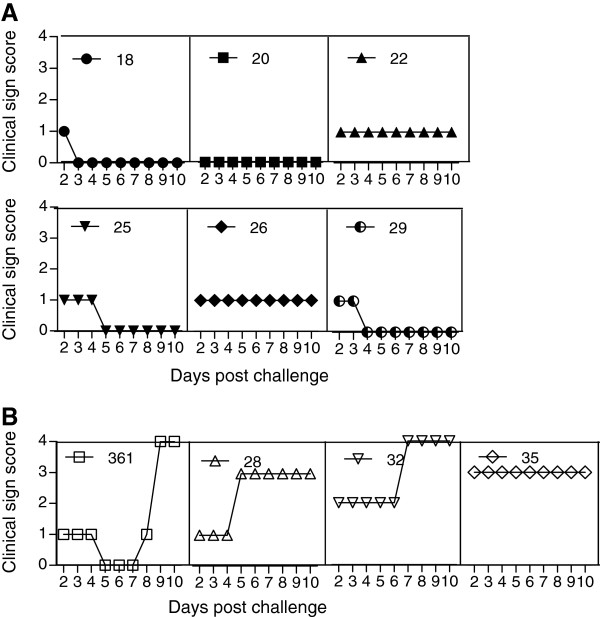
**Clinical scoring post-challenge with virulent FMDV.** Clinical signs of FMD rPPRV/VP1-inoculated goat (nos. 18, 20, 22, 25, 26, and 29) **(A)** and N75/1 –inoculated goat (nos. 361, 28, 32, and 35) **(B)** were observed from 2 to 10 days post challenge with FMDV JSL/06, and the clinical signs were scored as described in the Material and methods. Statistical differences were evaluated between two groups by the *t*-test method using GraphPad Prism statistical software, and the result showed the *p* < 0.01.

**Figure 6:**
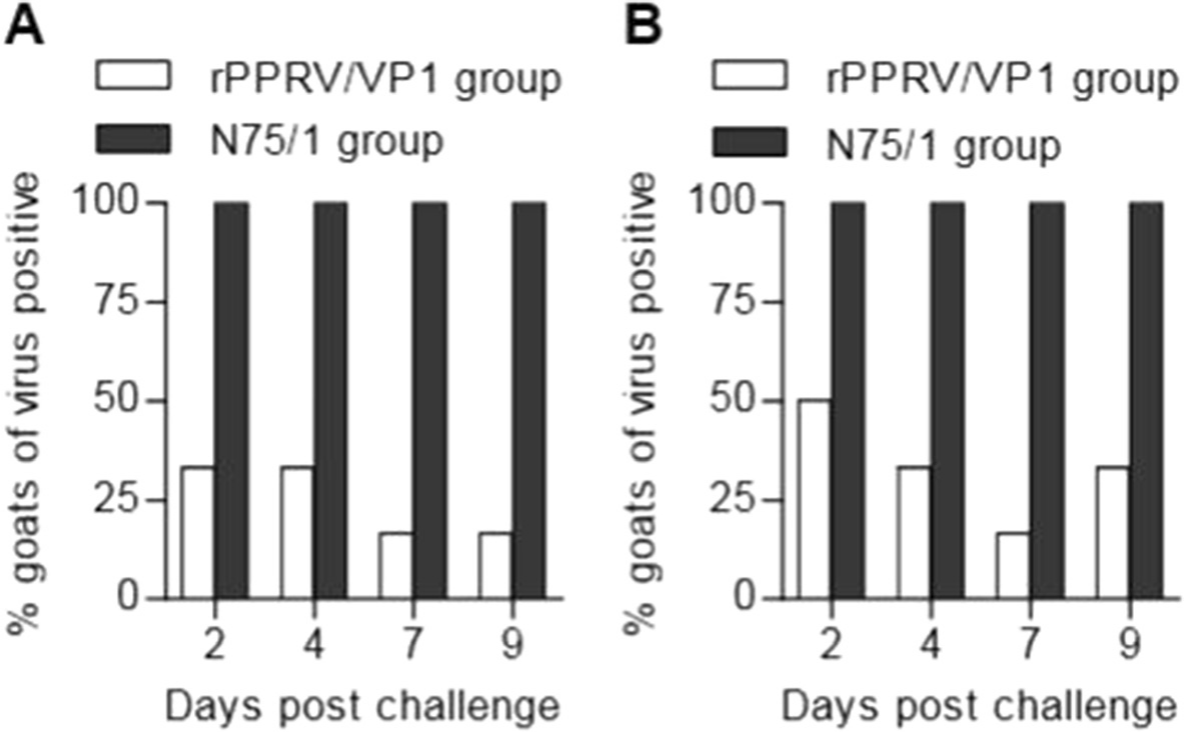
**Virusisolation post challenge.** Heparinized blood **(A)** and oropharyngeal swabs **(B)** for FMDV isolation were respectively collected from six individual rPPRV/VP1-inoculated goats and four individual N75/1-inoculated goats at 2, 4, 7, and 9 days post challenge with FMDV JSL/06. Viruses were isolated by inoculation of BHK-21 cell culture as described in the Material and methods.

To determine whether rPPRV/VP1 vaccination provided protective immunity against FMDV, the above six rPPRV/VP1-vaccinated goats and four N75/1-vaccinated goats were challenged with the virulent FMDV JSL/06. In the rPPRV/VP1-vaccinated group, no animal showed a notable change in body temperature after challenge. In contrast, all animals in the N75/1-vaccinated group developed fever by 2–4 days post-challenge (data not shown). Other than the FMDV inoculation point of one animal, no lesion was found on the oral mucosa or tongue surface in any rPPRV/VP1-infected animals post-challenge. However, vacuoles and/or ulcerated lesions were found in all N75/1-vaccinated animals post-challenge. Other than the FMDV inoculation points, water vacuoles or ulcerations were found on the tongue surface, gingiva, lips, and/or ungula of all control animals at different times 2–9 days post-challenge (data of photos were not shown). The severity of the lesions in each animal was quantitated using a scoring system based on clinical observation as described in the Material and methods section (Figure 5), which shows significant statistical differences in lesion scores between rPPRV/VP1- and N75/1-vaccinated animals (*p* < 0.01). Heparinized blood and oropharyngeal swab samples were also collected for FMDV isolation at 2, 4, 7, and 9 days post-challenge. Among six rPPRV/VP1-vaccinated animals, FMDV was recovered from the blood samples of 1–2 animals and from the oropharyngeal swabs of 1–3 animals at different detection times. However, FMDV was recovered from blood samples and oropharyngeal swabs of all four N75/1-vaccinated animals at different detection times (Figure 6). The results indicate that rPPRV/VP1 provided significant protection against challenge of virulent FMDV in goats.

## Discussion

In the present study, we generated the recombinant PPRV vaccine strain N75/1, which expressed the FMDV VP1 protein. This recombinant virus, rPPRV/VP1, induced 20–40 NA titer against FMDV and a comparable PPRV NA titer to that induced by N75/1 in goats. Moreover, rPPRV/VP1 provided significant protective immunity against FMDV challenge in goats. Our results suggest that rPPRV/VP1 is a potential dual, live-vectored vaccine against PPRV and FMDV.

The synthesis of VP1 gene was based on the sequence of FMDV isolate JSL/06 (GenBank accession no.: GU931682). The FMDV JSL/06 had been adapted in BHK-21 cells and is currently used as a master seed to manufacture inactivated vaccine for the controlling of FMDV Asia 1 in china. We sequenced the vp1 gene of the JSL/06 vaccine master seed and the JSL/06 challenge virus stock. There is no amino acid difference of VP1 between the vaccine seed and the challenge stock, and only one amino acid difference between the synthesized VP1 and that of the vaccine seed or the challenge stock.

We employed PPRV N75/1 as a live vaccine vector because it was shown to be highly immunogenic and induced a prolonged high level of PPRV NA in goats and sheep [8,17]. The rPPRV/VP1 induced an NA titer against PPRV that was comparable to that of N75/1 in goats and consistent with that of a previous report [22]. rPPRV/VP1 also showed a similar growth titer to the parent virus in Vero cells, which was different from that in a previous report regarding a recombinant rinderpest virus expressing partial sequences of the FMDV capsid VP1 protein [21].

A previous study had described that recombinant RPV expressing partial sequences of the FMDV capsid VP1 protein could not induce detectable neutralizing antibody against FMDV and provide poor protection against FMDV challenge [21]. For this reason, we vaccinated animals with a higher dosage (6 × 10^6^ TCID_50_) and got improved results in neutralizing antibody response and protection efficacy. Even though this dosage is too high for future field application, our study demonstrates that the strategy of PPRV vectored VP1 vaccine successfully works to induce detectable FMDV neutralizing antibody and provides significant protection against FMDV challenge, because the same antigen in a different animal species or with a different expression vector may result in a significantly different magnitude of immune response. In addition, the form of antigen expression might significantly impact the immune response; cytoplasmically expressed antigens generally have a bias towards presentation through the MHC-I pathway and therefore to induce a CD8+ T cell-mediated response. Secreted or cell membrane displayed antigen may induce more humoral immunity. In future studies, we will try to generate recombinant PPRV expressing the secreted VP1 or entire P1 and hope to get better neutralizing antibody responses with relative lower dosage. We will also try to test if repeat lower dose or combination use with whole virus inactivated vaccine (priming-boost) could improve antibody response and protective efficacy.

Compared to inactivated whole viral vaccine (FMDV NA titer is around 100) [30], rPPRV/VP1 elicited a relatively low level of FMDV NA (titer < 40) in goats, but provided significant protection from challenge of virulent FMDV; however, we can also get better immune effect through two dose vaccination, which could greatly increase the neutralizing antibody titer according to our previous study results [19,24,31]. Although the correlation between FMDV NA titer and protective efficacy in cattle has been well established [32-34], previous studies have shown that the protection against FMD is related not only to humoral immunity, but also to cellular immunity [35-37]. Previous studies have also shown that swine immunized with recombinant adenovirus expressing FMDV VP1 were completely protected against virulent FMDV challenge, even though the NA (titer < 40) induced by recombinant adenovirus expressing FMDV VP1 was significantly lower than that in swine inoculated with inactivated FMD vaccine [38,39]. Also, other reports showed that swine or cattle immunized with adenovirus expressing the FMDV P1 protein were protected against virulent FMDV challenge at post-vaccination day 7, although an NA titer was undetectable [28,40]. Although rPPRV/VP1 provided significant clinical protection, FMDV was still detected in blood samples or oropharyngeal swabs from partially immunized goats. Similar results were also observed in other studies with inactivated vaccines [4,41]. These results suggest that rPPRV/VP1 has a potential value for future application. In many countries, goats and sheep develop PPR, which results in huge economic losses and also presents a risk factor for FMD. The use of the live PPRV vaccine as a vector to develop recombinant vaccines against FMDV can control these two important diseases. Vaccination of rPPRV/VP1 provides clinical protection and probably decreases the FMDV transmission among goats or sheep.

To rapidly respond against future potential outbreaks, effective and emergent vaccinations should be developed as soon as possible. Currently, widely used inactivated whole viral FMD vaccine usually requires 7–10 days to develop protective immunity and induces only a short protective period [4,5,30]. A priming-boost regime presents an option to generate a rapid immune response and long-lasting protection. However, two doses of vaccination increases vaccination and labor costs. Also, the cost is increased because the inactivated FMD vaccine must be manufactured in a high-level biosafety facility. Using a PPRV vectored vaccine can prime the immune response to FMDV and may induce a long-lasting protection against FMD and PPR without the need for multiple doses of inactivated FMDV vaccine, thereby limiting the costs.

## Competing interests

ZB, WC, and CY are inventors on a pending patent application for the recombinant virus rPPRV/VP1. The remaining authors declare no financial or non-financial competing interests in the publication of this work.

## Authors’ contributions

CY performed the experimentation, including vaccination, challenge, and neutralizing antibody titer assay. WC performed the plasmid construction, rescue of the recombinant viruses, the immunofluorescence assay, western blotting and challenge, and contributed to the drafting of the manuscript. ZW, QH, XW, JG, and QY contributed to plasmid construction and rescue of the recombinant virus. HZ contributed to the vaccination and challenge experiments. CX provided general supervision. ZB designed the study, provided general supervision, and prepared the manuscript. All authors have read and approved the submission of the manuscript.
